# Selenoprotein R Protects Human Lens Epithelial Cells against d-Galactose-Induced Apoptosis by Regulating Oxidative Stress and Endoplasmic Reticulum Stress

**DOI:** 10.3390/ijms17020231

**Published:** 2016-02-10

**Authors:** Jie Dai, Hongmei Liu, Jun Zhou, Kaixun Huang

**Affiliations:** Hubei Key Laboratory of Bioinorganic Chemistry & Materia Medica, School of Chemistry and Chemical Engineering, Huazhong University of Science and Technology, 1037 Luoyu Road, Hongshan, Wuhan 430074, China; daijiehust@163.com (J.D.); hongmeiliuhust@hust.edu.cn (H.L.); hustzhj@hust.edu.cn (J.Z.)

**Keywords:** selenoprotein R, d-galactose, lens epithelial cells, apoptosis, oxidative stress, endoplasmic reticulum stress, mitochondrial membrane potential

## Abstract

Selenium is an essential micronutrient for humans. Much of selenium’s beneficial influence on health is attributed to its presence within 25 selenoproteins. Selenoprotein R (SelR), known as methionine sulfoxide reductase B1 (MsrB1), is a selenium-dependent enzyme that, like other Msrs, is required for lens cell viability. In order to investigate the roles of SelR in protecting human lens epithelial (hLE) cells against damage, the influences of *SelR* gene knockdown on d-galactose-induced apoptosis in hLE cells were studied. The results showed that both d-galactose and *SelR* gene knockdown by siRNA independently induced oxidative stress. When *SelR*-gene-silenced hLE cells were exposed to d-galactose, glucose-regulated protein 78 (GRP78) protein level was further increased, mitochondrial membrane potential was significantly decreased and accompanied by a release of mitochondrial cytochrome c. At the same time, the apoptosis cells percentage and the caspase-3 activity were visibly elevated in hLE cells. These results suggested that SelR might protect hLE cell mitochondria and mitigating apoptosis in hLE cells against oxidative stress and endoplasmic reticulum (ER) stress induced by d-galactose, implying that selenium as a micronutrient may play important roles in hLE cells.

## 1. Introduction

Selenium is an essential micronutrient for humans. Much of selenium’s beneficial influence on health is attributed to its presence within 25 selenoproteins [1,2,3,4,5]. Previous studies showed that supplementation of selenium could slow the development of naphthalene cataract, possibly by attenuating the oxidative stress in the lens [6]. However, the mechanism of selenium in preventing or slowing cataract onset and progression remains virtually unclear.

Oxidative stress is believed to play a major role in cataract formation. The research results [7,8] showed that the content of methionine sulfoxide (MetO) in membrane-bound protein of the lens increases with age, and in cataracts as much as 60% of the total membrane-bound protein methionine is found as MetO, suggesting a possible linkage between methionine oxidation and age-related cataracts. However, MetO can be reduced to Met by a class of enzymes known as methionine sulfoxide reductases (Msrs) [8,9]. Among Msrs, MsrB1 is the only selenoprotein, named as Selenoprotein R (SelR). SelR is widely distributed throughout different tissues [10] and localized in the cell nucleus and cytosol [11,12]. Because of the linkage between MetO and cataracts, SelR has received significant attention recently. Marchetti MA *et al*. proved that SelR, like other Msrs, is required for lens cell viability [13]. Our previous studies showed that SelR might play important roles in regulating redox homeostasis and attenuating ER stress induced by oxidative stress in human lens epithelial (hLE) cells [14,15,16].

d-Galactose (d-gal) is classified as a reducing monosaccharide. It is abundantly present in milk products and other non-dairy foodstuffs such as fruits and vegetables [17]. At normal concentrations it is metabolized into glucose, but at higher doses is converted into aldose and hydroperoxide through the action of d-galactose oxidase resulting in the formation of reactive oxygen species (ROS) [18]. d-Galactose-induced rat model of cataract has widely been investigated [19,20,21]. Three mechanisms involved in d-galactose-induced cataract have been proposed [21]. Oxidative stress is believed to play a pivotal role in cataract formation [22,23,24]. Several reports showed that oxidative damage contributed to d-galactose cataract development in immature and mature rats [25,26,27,28]. The lipid peroxide content was increased and gluthatione (GSH) content was decreased in lenses of male Wistar rats fed a d-galactose diet [28]. When the hLE cells were incubated with 125 mM d-galactose, the viability decreased and apoptosis increased and was accompanied by an increase of reactive oxygen species (ROS) level and decrease of GSH level [29]. However, the role of SelR protecting human lens epithelial cells against d-galactose-induced apoptosis has not been reported.

It is well known that damage to hLE cells is an early event in cataract development [22,23]. Cell apoptosis is suggested to be a crucial cause of cataract formation. It was reported that the incidence of apoptosis is greater in the LE cells of diabetic cataract rats and humans than that in those of non-diabetics [30]. Mulhern *et al.* [31] proved that in d-galactosemic rat lenses, mitotic LE cells in the central and peripheral mitotic zone died first, followed by the central, nonmitotic LE cells, and eventually the remaining LE cells.

In order to explore the nutritional role of selenium in a lens, in the present studies, we chose SelR as an example of 25 selenoproteins, used SRA01/04 cells, one kind of human lens epithelial cell line [32], as an experimental model and investigated the effect of *SelR* gene knockdown by RNAi on apoptosis in hLE cells. Oxidative stress, ER stress and mitochondrial dysfunctions associated with cell apoptosis were assayed. Our results suggest that SelR might protects hLE cells against d-galactose-induced apoptosis by inhibiting oxidative damage and ER stress via a mitochondrial apoptotic pathway, suggesting selenium as a micronutrient may play important roles in hLE cells.

## 2. Result

### 2.1. SelR Gene Silence Effectiveness

**Figure 1:**
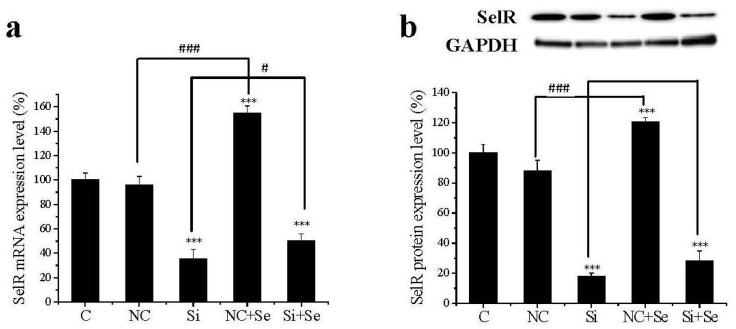
The efficiency of Selenoprotein R (*SelR*) gene knockdown in human lens epithelial (hLE) cells. *SelR* mRNA (**a**) and protein levels (**b**) in hLE cells were assayed by Real-time PCR and western blot using GAPDH as a reference. Data are the mean ± SD of at least three independent experiments. *** *p* < 0.001, compared to control group; ^#^ *p* < 0.05, ^###^ *p* < 0.001. C: control cells; NC: negative control celle; Si: *SelR* siRNA cells; NC+Se: negative control cells exposed to Na_2_SeO_3_ (1 μM) for 36 h; Si+Se: *SelR* siRNA cells exposed to Na_2_SeO_3_ (1 μM) for 36 h.

In order to evaluate the efficiency of *SelR* gene knockdown in hLE cells, levels of *SelR* mRNA and protein were determined before and after *SelR* siRNA transfection. The random siRNA as negative control did not affect the mRNA and protein expression levels of SelR. As shown in Figure 1, *SelR* mRNA (Figure 1a) and protein levels (Figure 1b) in *SelR* gene-silenced hLE cells were suppressed approximately 64.8% (*p* < 0.001) and 71.7% (*p* < 0.001), respectively, compared with normal control, showing that the expression of *SelR* was successfully depressed by siRNA. Influence of Na_2_SeO_3_ on the expression of SelR in hLE cells was also analyzed. *SelR* mRNA (Figure 1a) and protein (Figure 1b) expression in cells treated with Na_2_SeO_3_ (1 μM) were increased 58.8% and 34.0%, respectively, compared with the negative control. When hLE cells were treated with *SelR* siRNA, *SelR* mRNA and protein expression in cells exposed with Na_2_SeO_3_ (1 μM) were increased 15.1% and 8.8%, respectively, compared with the *SelR* siRNA group.

### 2.2. Effect of SelR Gene Knockdown and Na_2_SeO_3_ on Cell Viability in d-Galactose-Treated hLE Cells

**Figure 2:**
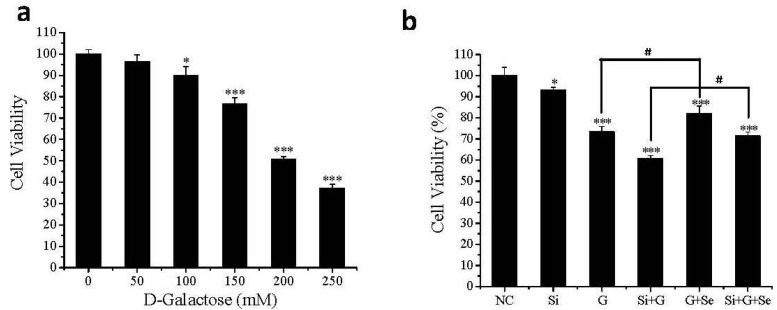
Effect of *SelR* gene knockdown and Na_2_SeO_3_ on d-galactose-induced cell death. (**a**) The viability of hLE cells after treatment with the indicated concentrations of d-galactose; (**b**) The viability of hLE cells in the indicated groups. Data are the mean ± SD of at least three independent experiments. * *p* < 0.05, *** *p* < 0.001, compared to the negative control group; # *p* < 0.05. NC: negative control cells; Si: cells with *SelR* siRNA transfection for 24 h; G: cells exposed to d-galactose (150 mM) for 36 h; Si+G: cells with *SelR* siRNA transfection followed by d-galactose exposure; G+Se: cells exposed to galactose (150 mM) and Na_2_SeO_3_ (1 μM) for 36 h; Si+G+Se: cells with *SelR* siRNA transfection followed by galactose and Na_2_SeO_3_ exposure.

The effect of *SelR* gene knockdown by RNAi on d-galactose-induced hLE cells death was investigated using the MTT assay. As shown in Figure 2a, the viability of cells was significantly decreased in a concentration-dependent manner. After the incubation with 50, 100, 150, 200 and 250 mM d-galactose for 36 h, cell viabilities were 96.36%, 90.01%, 76.56% (*p* < 0.001), 50.74% (*p* < 0.001) and 37.13% (*p* < 0.001) of untreated cells, respectively. Effect of *SelR* gene knockdown and Na_2_SeO_3_ on d-galactose-induced cell viabilities was shown in Figure 2b. The viabilities of *SelR*-gene-silenced cells (Si group), cells treated with 150 mM d-galactose for 36 h (G group) and *SelR*-gene-silenced cells followed with d-galactose treatment (Si+G group) were approximately 93.10%, 73.40% (*p* < 0.001) and 60.63% (*p* < 0.001) of negative control, respectively. When hLE cells treated with d-galactose (150 mM) were cultivated with Na_2_SeO_3_ (1 μM) for 36 h, the viabilities of G+Se group and Si+G+Se group were increased by 8.5% and 10.7% , respectively, compared to G group and Si+G group.

### 2.3. Effect of SelR Gene Knockdown and Na_2_SeO_3_ on d-Galactose-Induced Cell Apoptosis

**Figure 3:**
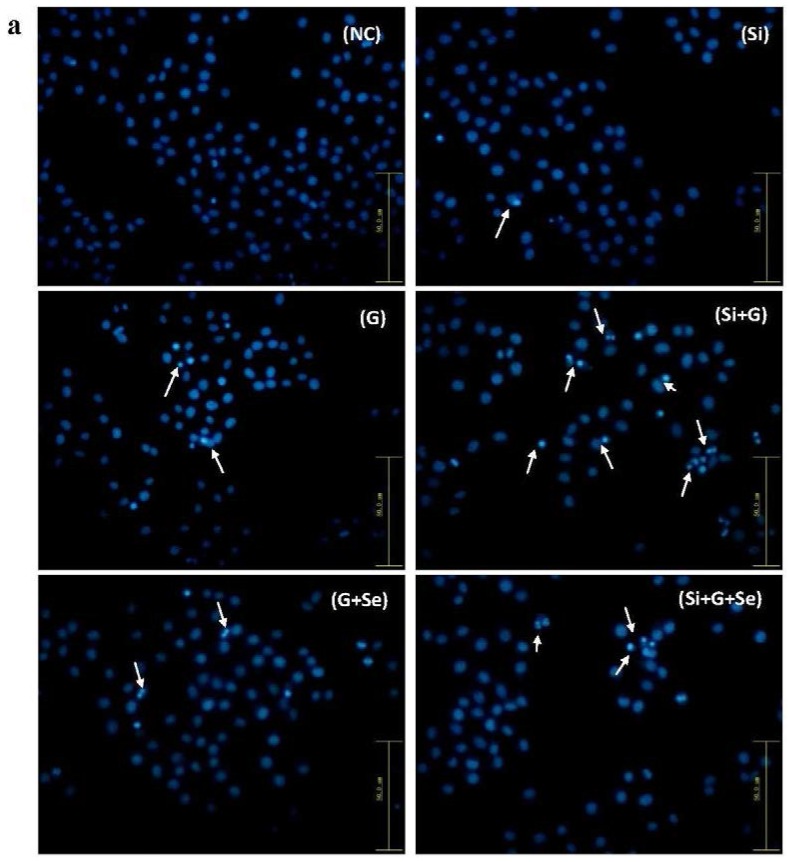

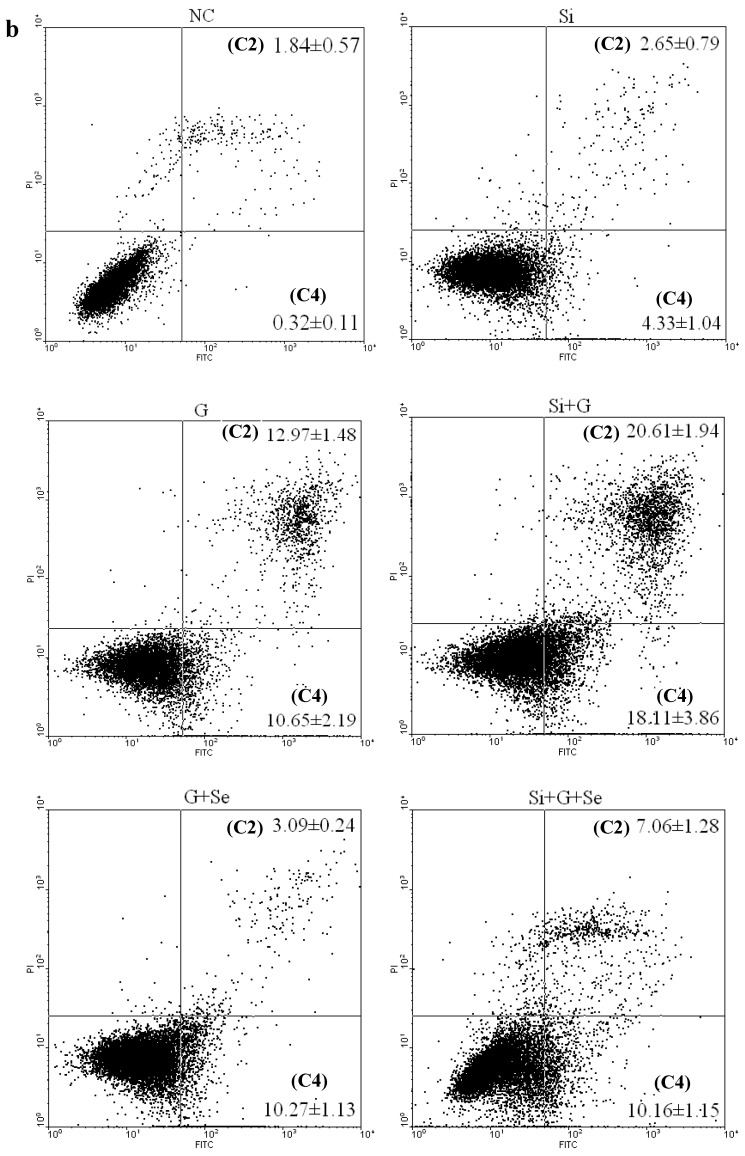

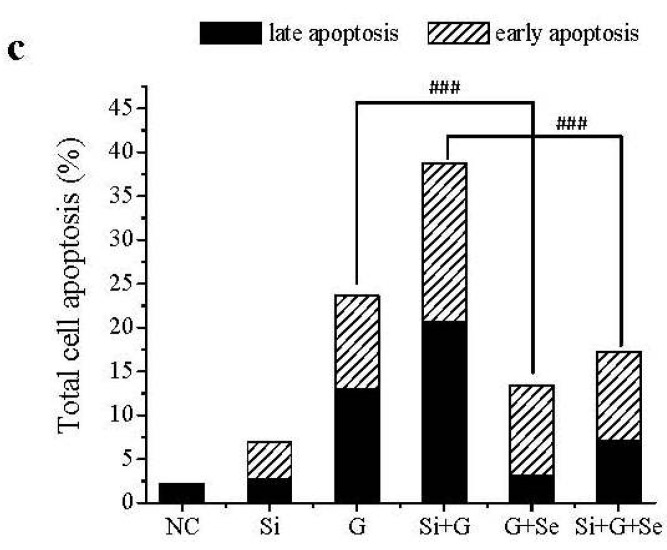
Effect of *SelR* gene knockdown and Na_2_SeO_3_ on d-galactose-induced cell apoptosis. (**a**) hLE cell morphological changes in the indicated groups under the fluorescence microscopy after staining with Hoechst 33258 (200×); (**b**) Quantitative analysis of hLE cells apoptosis using flow cytometry by an Annexin-V-FITC apoptosis detection kit; (**c**) Total apoptosis cell percentage in the indicated groups. The black body and oblique line represent number of the late apoptosis cells (C2) and early apoptosis cells (C4). Data are the mean ± SD of at least three independent experiments. Arrows, examples of apoptotic cells. ^###^ *p* < 0.001. NC: negative control cells; Si: cells with *SelR* siRNA transfection for 24 h; G: cells exposed to d-galactose (150 mM) for 36 h; Si+G: cells with *SelR* siRNA transfection followed by d-galactose exposure; G+Se: cells exposed to d-galactose (150 mM) and Na_2_SeO_3_ (1 μM) for 36 h; Si+G+Se: cells with *SelR* siRNA transfection followed by d-galactose and Na_2_SeO_3_ exposure.

Morphological changes of cell nuclei were observed using a fluorescence microscope by staining with Hoechst 33258 (Figure 3a). As shown in Figure 3a, the negative control hLE cells nucleus remained uniformly stained (Figure 3a (NC)). After treatment with 150 mM d-galactose, a typical apoptotic morphology was visible in some cells (Figure 3a (G)). When *SelR*-gene-silenced cells were treated with 150 mM d-galactose, the cells exhibited obvious nuclear condensation and fragmentation (Figure 3a (Si+G)). When hLE cells treated with d-galactose (150 mM) were cultivated with Na_2_SeO_3_ (1 μM) for 36 h, a decreased number of cells exhibiting fragmented nuclei were observed in G+Se group and Si+G+Se group, compared to G group and Si+G group.

Quantitative analysis of cell apoptosis was carried out using flow cytometry. As shown in Figure 3, the early apoptotic and late apoptotic fraction, respectively, was 1.84% and 0.32% in negative control cells (Figure 3b (NC)); 2.65% and 4.33% in cells transfected with *SelR* siRNA (Figure 3b (Si)); 12.97% and 10.65% in cells exposed to d-galactose (Figure 3b (G)); 20.61% and 18.11% in cells transfected with *SelR* siRNA followed by d-galactose stimulation (Figure 3b (Si+G)). When hLE cells were treated with *SelR* siRNA followed by d-galactose and Na_2_SeO_3_ treatment, the early and late apoptotic fraction was 7.06% and 10.16% (Figure 3b (Si+G+Se)). Compared to G group and Si+G group, the total apoptosis cell percentage was decreased to 0.57-fold and 0.45-fold in G+Se group and Si+G+Se group, respectively (Figure 3c). Seleniun supplementation may obviously decrease the percentage of apoptosis induced by d-galactose or *SelR* siRNA and d-galactose in hLE cells.

### 2.4. Effect of SelR Gene Knockdown and Na_2_SeO_3_ on GPx Activity in d-Galactose-Treated hLE Cells

**Figure 4:**
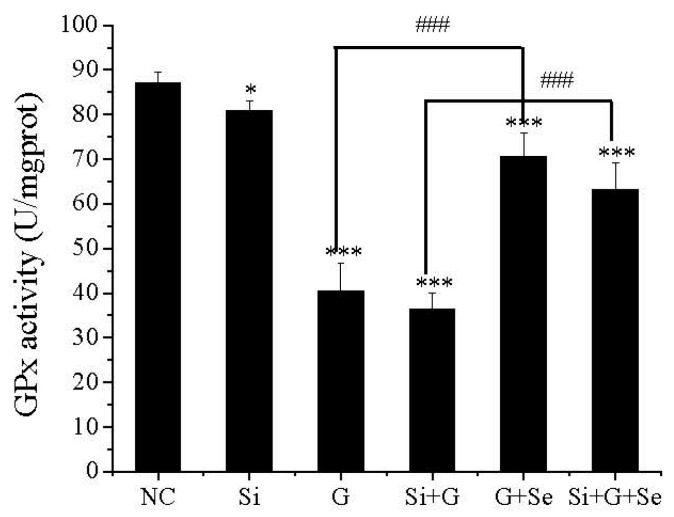
Alterations of intracellular glutathione peroxidase 1 (GPx) activity. Data are the mean ± SD of at least three independent experiments. * *p* < 0.05, *** *p* < 0.001, compared to the negative control group; ^###^ *p* < 0.001. NC: negative control cells; Si: cells with *SelR* siRNA transfection for 24 h; G: cells exposed to d-galactose (150 mM) for 36 h; Si+G: cells with *SelR* siRNA transfection followed by d-galactose exposure; G+Se: cells exposed to d-galactose (150 mM) and Na_2_SeO_3_ (1 μM) for 36 h; Si+G+Se: cells with *SelR* siRNA transfection followed by d-galactose and Na_2_SeO_3_ exposure.

As shown in Figure 4, compared to negative control cells (NC), GPx activity was decreased to 92.9%, 46.5% and 41.6%, respectively, in *SelR*-gene-silenced cells (Si), d-galactose-exposed cells (G) and cells transfected with *SelR* siRNA followed by d-galactose-exposure (Si+G). When hLE cells treated with d-galactose (150 mM) were cultivated with Na_2_SeO_3_ (1 μM) for 36 h, GPx activity was increased to approximately 1.75- and 1.74-fold in G+Se group and Si+G+Se group, compared to G group and Si+G group.

### 2.5. Effect of SelR Gene Knockdown and Na_2_SeO_3_ on d-Galactose-Induced ROS, MDA, PC and TSH Levels

**Figure 5:**
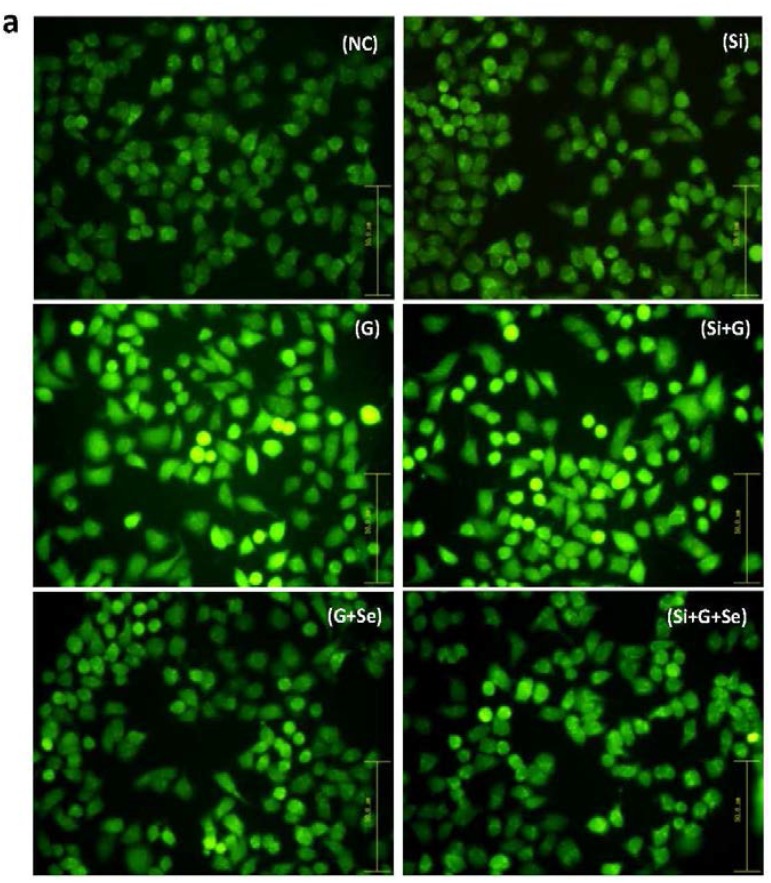

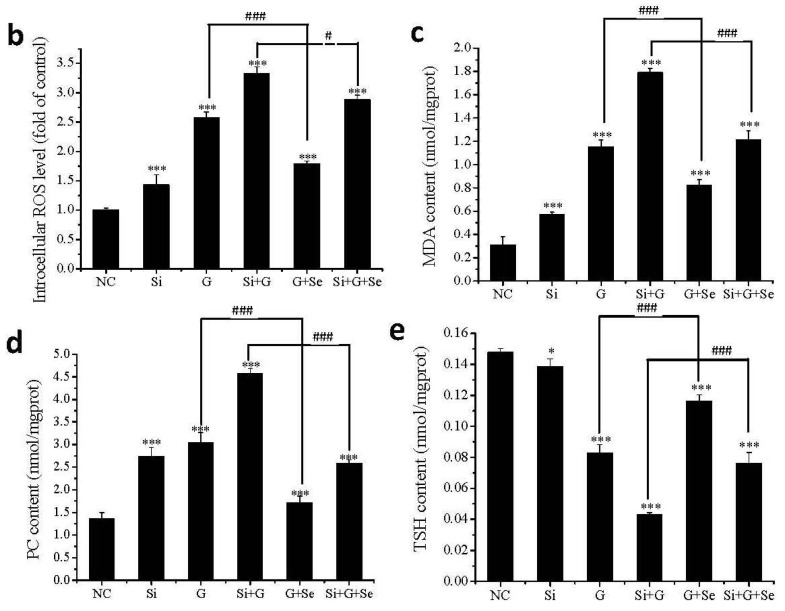
Alterations of intracellular reactive oxygen species (ROS), malondialdehyde (MDA), protein carbonyl (PC) and total mercapto group (TSH) levels. (**a**) Alterations of fluorescent intensity with ROS levels in the indicated group assayed by H_2_-DCFDA staining using fluorescent microscopy (200×); (**b**) Intracellular ROS levels in hLE cells in the indicated group; (**c**) Intracellular MDA levels in hLE cells in the indicated group; (**d**) Intracellular PC levels in hLE cells in the indicated group; (**e**) Intracellular TSH levels in hLE cells in the indicated group. Data are the mean ± SD of at least three independent experiments. * *p* < 0.05, *** *p* < 0.001, compared to the negative control group; ^#^ *p* < 0.05, ^###^ *p* < 0.001. NC: negative control cells; Si: cells with *SelR* siRNA transfection for 24 h; G: cells exposed to d-galactose (150 mM) for 36 h; Si+G: cells with *SelR* siRNA transfection followed by d-galactose exposure; G+Se: cells exposed to d-galactose (150 mM) and Na_2_SeO_3_ (1 μM) for 36 h; Si+G+Se: cells with *SelR* siRNA transfection followed by d-galactose and Na_2_SeO_3_ exposure.

ROS generation was measured using fluorescence microscopy (Figure 5a). The fluorescent intensity of ROS was enhanced in the cells treated with *SelR* SiRNA (Figure 5a (Si)) and in the cells exposed to d-galactose (Figure 5a (G)), compared to negative control cells (Figure 5a (NC)). The intensity was further enhanced in *SelR*-gene-silenced cells exposed to d-galactose (Figure 5a (Si+G)). Meanwhile, intracellular ROS levels were quantified by flow cytometry using DCFH-DA fluorescent probes. As shown in Figure 5b, compared to negative control cells (NC), intracellular ROS level was increased to 1.43-fold, 2.57-fold and 3.33-fold, respectively, in *SelR*-gene-silenced cells (Si), d-galactose-exposed cells (G) and cells transfected with *SelR* siRNA followed by d-galactose-exposure (Si+G). When hLE cells treated with d-galactose (150 mM) were cultivated with Na_2_SeO_3_ (1 μM) for 36 h, ROS level was decreased to approximately 0.69- and 0.86-fold in G+Se group and Si+G+Se group, compared to G group and Si+G group.

Influence of *SelR* gene knockdown and Na_2_SeO_3_ on MDA content in hLE cells was also analyzed. As shown in Figure 5c, MDA level in *SelR* gene knockdown group was 1.84-fold (0.57 nmol/mgprot) of that in control group, after treatment with 150 mM d-galactose, MDA content was increased to approximately 3.71.-fold (1.15 nmol/mgprot) and 5.77-fold (1.79 nmol/mgprot) before and after *SelR* gene knockdown, respectively, compared with untreated control cells. When hLE cells were treated with d-galactose (150 mM) and Na_2_SeO_3_ (1 μM) for 36 h, MDA level was decreased to approximately 0.71-fold (0.82 nmol/mgprot) and 0.68-fold (1.21 nmol/mgprot) in G+Se group and Si+G+Se group, compared to G group or Si+G group.

Furthermore, the changes of PC content in these groups were almost the same as changes of ROS and MDA generation. As shown in Figure 5d, PC level in *SelR* gene knockdown group was 2.01-fold (2.73 nmol/mgprot) of that in negative control group; after treatment with 150 mM d-galactose, PC content was increased to approximately 2.23-fold (3.04 nmol/mgprot) and 3.36-fold (4.57 nmol/mgprot) before and after *SelR* gene knockdown, respectively, compared with negative control cells. When hLE cells were treated with d-galactose (150 mM) and Na_2_SeO_3_ (1 μM) for 36 h, PC level was decreased to approximately 0.56-fold (1.71 nmol/mgprot) and 0.56-fold (2.58 nmol/mgprot) in G+Se group and Si+G+Se group, compared to G group or Si+G group.

Sulfhydryl groups represent the level of antioxidants in tissue and are preferentially consumed by free radicals [33]. As shown in Figure 5e, TSH level in *SelR* gene knockdown group was 93.9% (0.139 nmol/mgprot) of that in negative control group; after treatment with 150 mM d-galactose, PC content was decreased to approximately 56.1% (0.083 nmol/mgprot) and 29.1% (0.043 nmol/mgprot) before and after *SelR* gene knockdown, respectively, compared with negative control cells. When hLE cells were treated with d-galactose (150 mM) and Na_2_SeO_3_ (1 μM) for 36 h, TSH level was increased to approximately 1.39-fold (0.116 nmol/mgprot) and 1.77-fold (0.076 nmol/mgprot) in G+Se group and Si+G+Se group, compared to G group and Si+G group.

### 2.6. Effect of *SelR* Gene Knockdown on d-Galactose-Induced Mitochondrial Dysfunctions in hLE Cells

**Figure 6:**
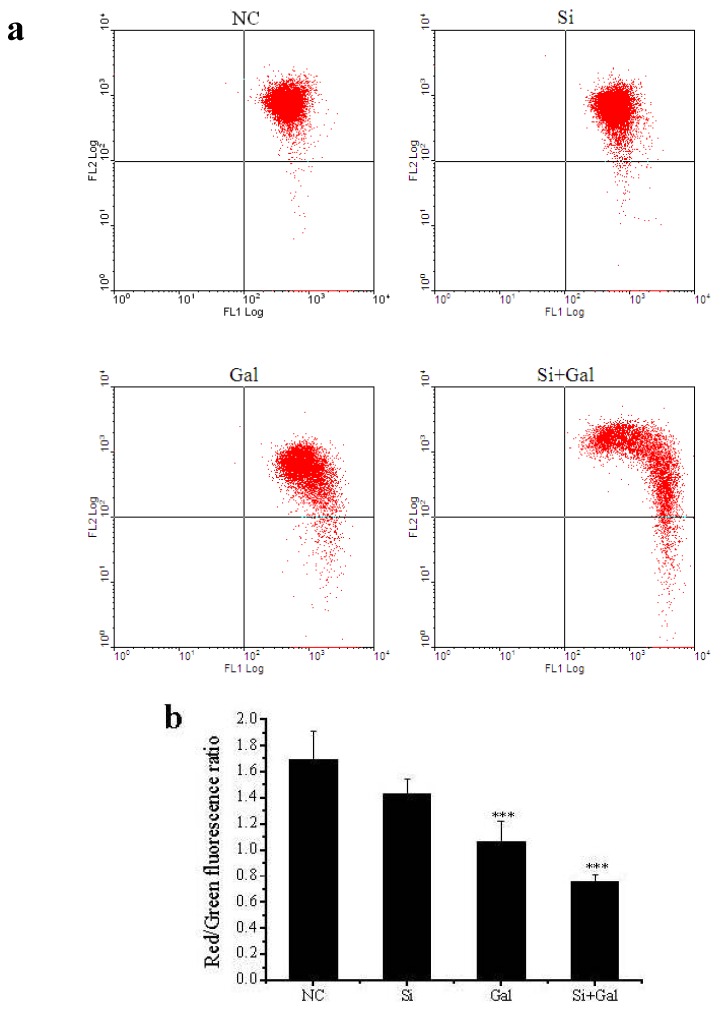
Quantitative analysis of Δψm using flow cytometry in hLE cells. (**a**) Changes of mitochondrial membrane potential (Δψm) in hLE cells; (**b**) Δψm was quantified using the ratio of red to green fluorescence. Data are the mean ± SD of at least three independent experiments. *** *p* < 0.001, compared to the negative control group. NC: negative control cells; Si: cells with *SelR* siRNA transfection for 24 h; Gal: cells exposed to d-galactose (150 mM) for 36 h; Si+Gal: cells with *SelR* siRNA transfection followed by d-galactose exposure.

To investigate the effects of *SelR* gene knockdown or/and d-galactose treatment on mitochondrial function, mitochondrial transmembrane potential Δψm was measured. As shown in Figure 6, the Δψm value in *SelR*-gene-silenced hLE cells was 88% of that in negative control cells. When the cells were treated with 150 mM d-galactose, the Δψm was decreased to approximately 63% (*p* < 0.001) of normal cell; when *SelR*-gene-silenced hLE cells were exposed 150 mM d-galactose, the Δψm was decreased to approximately 45% (*p* < 0.001) of the negative control cell.

### 2.7. Effect of SelR Gene Knockdown on Cytochrome c Release in d-Galactose-Treated hLE Cells

**Figure 7:**
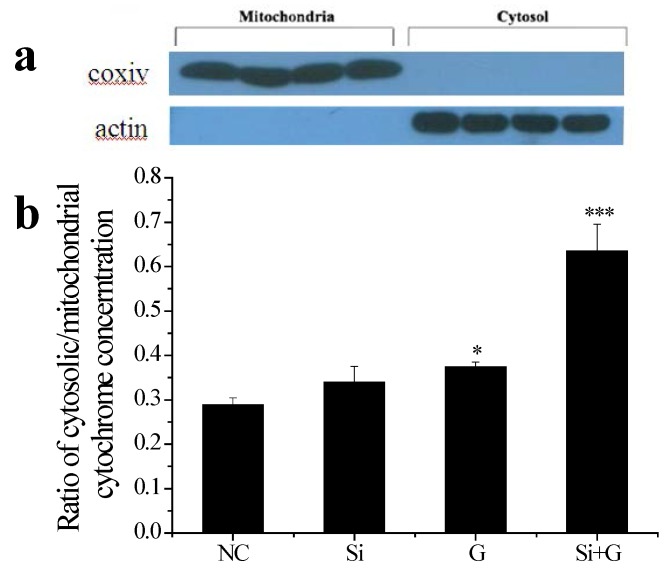
Effect of *SelR* gene knockdown on d-galactose-induced cytochrome c release. (**a**) Purity of cytosolic and mitochondrial fractions; (**b**) ELISA quantization of cytochrome c protein in cytosolic and mitochondrial fractions. The data are expressed as the ratio of cytosolic-to- mitochondrial cytochrome c concentrations. * *p* < 0.05, *** *p* < 0.001, compared to the negative control group. NC: negative control cells; Si: cells with *SelR* siRNA transfection for 24 h; G: cells exposed to d-galactose (150 mM) for 36 h; Si+G: cells with *SelR* siRNA transfection followed by d-galactose exposure.

The negative effects of *SelR* gene knockdown or d-galactose on mitochondrial function were demonstrated. As shown in Figure 7a, Mitochondrial and cytosolic fractions were separated using a mitochondria/cytosol fractionation kit. The treatments increasd the ratio of cytosolic-to-mitochondrial cytochrome c concentrations, with values of 0.29 (NC), 0.34 (Si), 0.37 (G), 0.64 (Si+G), respectively (Figure 7b).

### 2.8. Effect of SelR Gene Knockdown on Caspase-3 Activity in d-Galactose-Treated hLE Cells

**Figure 8:**
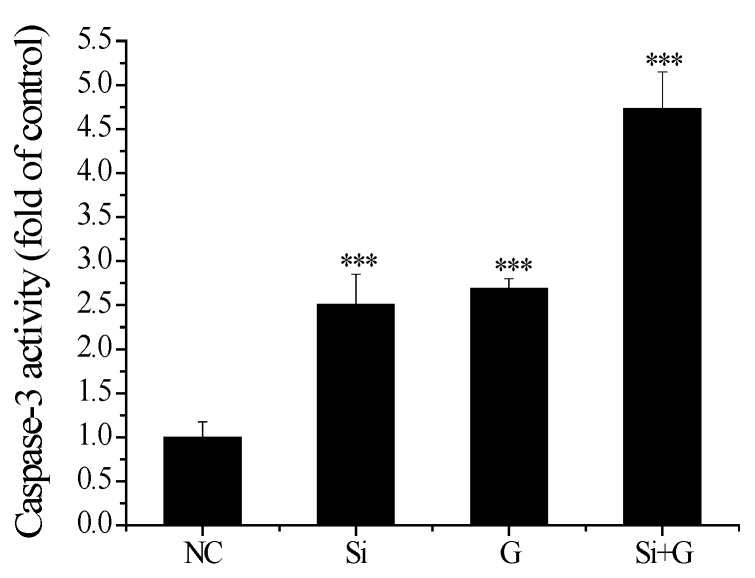
Effect of *SelR* gene knockdown on d-galactose-induced caspase-3 activity. Data are the mean ± SD of at least three independent experiments. *** *p* < 0.001, compared to the negative control group. NC: negative control cells; Si: cells with *SelR* siRNA transfection for 24 h; G: cells exposed to d-galactose (150 mM) for 36 h; Si+G: cells with *SelR* siRNA transfection followed by d-galactose exposure.

To understand the pathway of d-galactose -induced hLE cells apoptosis, we analyzed caspase-3 activity. As shown in Figure 8, caspase-3 activities were increased after *SelR* gene knockdown or gactose exposure, with values of 2.51-fold (*p* < 0.001) and 2.69-fold (*p* < 0.001) of untreated cells group, respectively. In *SelR*-gene-silenced cells followed by 150 mM d-galactose treatment, the caspase-3 activity was 4.73-fold (*p* < 0.001) higher compared to negative control hLE cells (Figure 8).

### 2.9. Effect of SelR Gene Knockdown on GRP78 Protein Expression in Glactose-Treated hLE Cells

**Figure 9:**
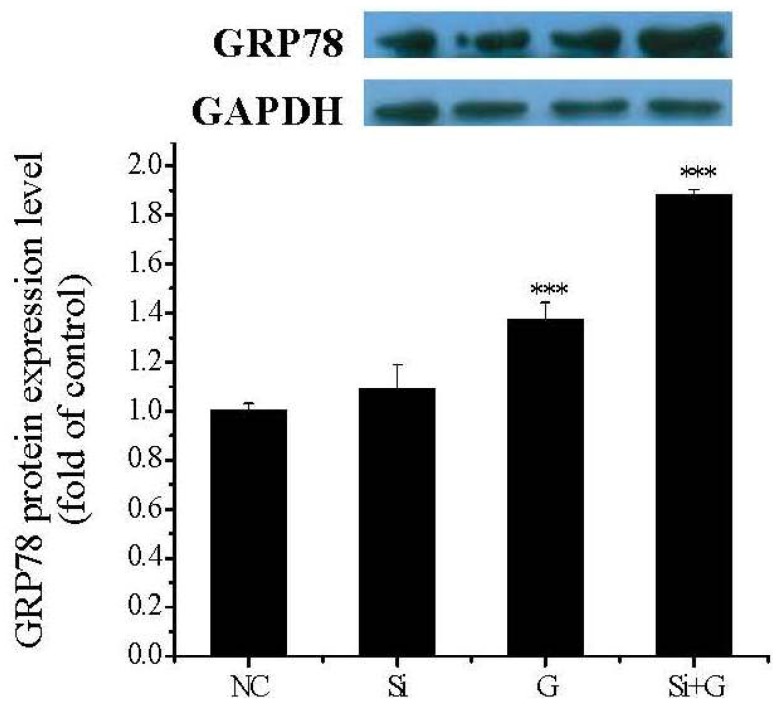
Effect of *SelR* gene knockdown on d-galactose-induced GRP78 protein expression. Data are the mean ± SD of at least three independent experiments. *** *p* < 0.001, compared to the negative control group. NC: negative control cells; Si: cells with *SelR* siRNA transfection for 24 h; G: cells exposed to d-galactose (150 mM) for 36 h; Si+G: cells with *SelR* siRNA transfection followed by d-galactose exposure.

To inspect the effect of *SelR* gene knockdown or galactose exposure on ER stress, GRP78 protein expression was investigated by western blot in this study. As shown in Figure 8, GRP78 protein level was increased after d-galactose exposure, with value of 1.37-fold (*p* < 0.001) of negative control cells. In *SelR*-gene-silenced cells followed by 150 mM d-galactose treatment, the GRP78 protein level was 1.88-fold (*p* < 0.001) higher compared to negative control cells (Figure 9).

## 3. Discussion

The lens of the vertebrate eye contains a single layer of epithelial cells on its anterior surface [34], which is important for maintaining the metabolic homeostasis and transparency of the entire lens [35]. It was reported that lens epithelial cell apoptosis might be a common cellular basis for initiation of noncongenital cataract formation [30]. The incidence of apoptosis in the LE cells of diabetic cataract rats and humans was greater than that in those of non-diabetics [36]. Previous data showed that high-d-galactose concentrations significantly inhibited the percentage of hLE cells survival at 24–72 h incubation [37]. Consistently, we observed in this study that high-d-galactose concentrations could significantly increase SRA01/04 cells death and apoptosis (Figure 2 and Figure 3). When *SelR*-gene-silenced SRA01/04 cells were exposed to d-galactose, the viabilities were further decreased in different concentrations of d-galactose (Figure 2). Those results suggest that SelR may decrease hLE cells apoptosis induced by d-galactose, although this role of SelR needs to be proved *in vivo*.

Oxidative stress has long been recognized as an important stimulus of apoptosis in lens epithelial cells and also plays a pivotal role in the pathogenesis of cataracts. Several reports showed that oxidative stress is a prime cause of diabetic complications and high d-galactose could induce excessive ROS production, promote lipid peroxidation and inhibit cell proliferation in various types of cells [14,38,39,40] and that oxidative stress-induced ER stress could lead to apoptosis [12,41,42,43,44]. In the present study, the decrease of hLE cells viabilities was related to the significant increase of ROS, MDA and PC levels (Figure 5) induced by d-galactose; furthermore, the GRP78 protein level was significantly increased in d-galactose-stimulated hLE cells (Figure 9), indicating that ER stress occured. When the hLE cells treated by *SelR* SiRNA were exposed to d-galactose, GRP78 protein level was further increased; meanwhile, there was a similar increase for the percentage of apoptosis cells. These results imply that SelR plays important roles in protecting hLE cells against apoptosis by regulating redox balance and inhibiting ER stress resulted from d-galactose-induced oxidative stress in hLE cells.

In order to extend our knowledge of SelR protecting hLE cells by regulating redox balance, the integrity of the mitochondrial membrane potential (Δψm) was evaluated and release of cytochrome c from mitochondria was measured. It is well known that are both a source and target of ROS. Oxidation of mitochondrial pores by cytosolic ROS is one of the major reasons for the release of cytochrome c due to disruption of the mitochondrial membrane potential Δψm [45]. SelR, which contains selenocysteine and is a selenoprotein localized in the cell nucleus and cytosol, has the highest Msr activity [46] and might repair cytosolic proteins by converting oxidized Met residues (R-MetO) to Met directly [12]. In present study, our results show that when the cells were only treated with *SelR* knockdown by siRNA, mitochondrial dysfunction occurred; when *SelR*-gene-silenced cells were exposed to d-galactose (150 mM) for 36 h, mitochondrial dysfunction was further exacerbated, and the decrease of Δψm was accompanied (Figure 6) by an increased ratio of cytosolic-to-mitochondrial cytochrome c concentrations (Figure 7), meanwhile with an increased activity of caspase-3 (Figure 8), indicating a role of SelR in protection mitochondria against d-galactose-induced mitochondrial damage in hLE cells. These results further support our previous speculation [14] that oxidized methionine residues in proteins repaired by SelR may continue to react with ROS. Thus, SelR indirectly scavenges ROS in cytosol. On the other hand, the –SeH group of selenocysteine with the strong reducibility in SelR may directly react with ROS in cytosol to reduce the damage of cytosolic ROS to mitochondria. Conversely, when the *SelR* gene was knockdown by RNAi, SelR protein expression decreased, proteins containing R–MetO were not repaired and ROS were not scavenged, oxidative stress turns up. Oxidative stress has been demonstrated as being able to cause the burst of ROS from mitochondria which in turn induce the loss of Δψm [45]. When ROS accumulation reaches a critical threshold and cell’s mitochondrial damage occurs, the cytochrome c from the mitochondria releases to cytosol, and then activates caspases apoptotic pathway [47]. As expected, our data show that treatment by only knockdown the *SelR* gene using siRNA or high d-galactose resulted in oxidative damage (Figure 5), an increase in cell apoptosis (Figure 3) and a decrease in cell viability (Figure 2). The present data suggest that oxidative stress plays a pivotal role in d-galactose cataract formation and SelR plays important role in protecting mitochondria against d-galactose-induced oxidative damage.

However, SelR is not only a selenoprotein in hLE cells, such as glutathione peroxidase 1 (GPx1). Flohé [5] showed that the lack of GPx-1 due to alimentary selenium deprivation was inferred to induce cataracts in rats and to cause cataracts in mice by targeted gene disruption. In the present study, our results showed that selenium supplementation could significantly decrease the percentage of apoptosis induced by d-galactose or *SelR* siRNA and d-galactose in hLE cells (Figure 3). When the *SelR* gene underwent knockdown by siRNA, selenium supplementation may have increased other selenoprotein expression; for example, GPx1 activity was elevated (Figure 4). It is clear from these results that the role of selenium protecting hLE cells against apoptosis by regulating redox balance is not only via SelR. Therefore, it is necessary to do more studies on the nutritional biochemistry of selenium in lenses.

## 4. Materials and Methods

### 4.1. Materials

Newborn calf serum (NCS), Dulbecco’s Modified Eagle’s Medium (DMEM), and 3-(4,5-dimethylthiazol-2-yl)-2,5-diphenylte-trazolium bromide (MTT) were purchased from Gibco BRL (Gaithersburg, MD, USA); streptomycin sulfate and Penicillin G were purchased from Amersco (Cochran, GA, USA). Lipofectamine 2000 was obtained from Invitrogen (Camarillo, CA, USA). d-Galactose and protease inhibitor cocktail were purchased from Sigma Co. (Santa Clara, CA, USA) All other chemicals were of the highest commercial grade available.

### 4.2. Cell Culture

hLE cells were divided into six groups: negative control cells (NC), d-galactose-exposed cells (G), *SelR*-gene-silenced cells (Si), *SelR*-gene-silenced cells followed with d-galactose exposure for 36 h (Si+G), cells exposed to d-galactose (150 mM) and Na_2_SeO_3_ (1 μM) for 36 h (G+Se) and cells with *SelR* siRNA transfection followed by d-galactose and Na_2_SeO_3_ exposure (Si+G+Se). hLE cells (SRA01/04) were maintained in DMEM supplemented with 10% heat-inactivated newborn calf serum and antibiotics (penicillin G 100 U/mL, streptomycin 100 μg/mL) at 37 °C in the presence of 5% CO_2_. NaHCO_3_ of 0.37% was supplemented in NC and Si groups, 0.1% in other groups.

In order to detect influence of different osmolarity to cell, we added 75 mM NaCl in medium to match the increased osmolarity of the 150 mM galactose for the 36 h and then evaluated the effect of supplemented NaCl on cell viability (Figure S1A) and cell apoptosis (Figure S1B). The results showed that there were not significant changes on cell viability and cell apoptosis in NaCl group compared to normal control group.

### 4.3. SelR RNA Interference and Cell Treatment

Double-stranded short interfering RNAs (siRNA) specific for *SelR* were 5′-GCGUCCGGAGCACAAUAGATT-3′ (sense) and 5′-UCUAUUGUGCUCCGGACGCTT-3′ (antisense) [13], and 5′-UUCUCCGAACGUGUCACGUTT-3′ (sense) and 5′-ACGUGACACGUUCGGAGAATT-3′ (antisense) for negative control. siRNAs were synthesized by Shanghai GenePharma (Shanghai, China).

Cells of the Si, Si+G and Si+G+Se groups were transfected with siRNA as described in previous works [48]. After 24 h, all groups were treated with fresh serum-free media with or without d-galactose (150 mM) and Na_2_SeO_3_ for 36 h. hLE cells were then harvested for further analysis.

### 4.4. Cell Viability Assay

Cell viability was measured by MTT assay [49]. The absorbance was measured at 570 nm using a spectrophotometer (Cecil, Nottingham Model No. CE7200; Cambridge, UK) and expressed as percent of the absorbance in untreated control cells in parallel wells [46].

### 4.5. Isolation of Total RNA and Real-Time PCR

According to the manufacturer’s instruction, total RNAs were extracted from the hLE cells using Trizol reagent (Invitrogen). cDNA was obtained by incubation of the mRNA with M-MLV reverse transcriptase (Toyobo, Osaka, Japan), oligo (dT) (Toyobo) and dNTPs (Toyobo) at 42 °C for 40 min in the buffer (Toyobo). After inactivation of the enzyme by incubation at 95 °C for 5 min, polymerase chain reaction (PCR) was carried. The primer sequences are as follow: *SelR*: 5′-ATGTCGTTCTGCAGCTTCTTC-3′ (forward) and 5′-CACACTTGCCACAGGACAC-3′ (reverse) [50]; GAPDH: 5′-CCATGTTCGTCATGGGTGTGAACCA-3′ (forward) and 5′-GCCAGTAGAGGCAGGGATGATGTTC-3′ (reverse) [51]; Real-time PCR was performed in DNA Engine Opticon 2 (MJ Research, Watertown, MA, USA). PCR conditions for *SelR* were 95 °C for 10 min followed by 40 cycles of 95 °C for 15 s and 60 °C for 1 min; for GAPDH, the conditions were referred [51].

### 4.6. Western Blot Analysis

Western blots were processed as described in previous work [51,52]. Briefly, hLE cells (~1 × 10^7^ cells per sample) were lysed on ice for 20 min in westen lysis buffer and protein extracts were obtained by centrifugation (4 °C, 12,000× *g* for 15 min). Protein concentration was analyzed using the Bradford assay. After SDS-PAGE and transferred electrophoretically to polyvinylidene fluoride (PVDF) membranes, the membranes were blocked for 120 min at room temperature with 5% (*w*/*v*) non-fat milk. Then the blots was incubated overnight at 4 °C with 5% non-fat milk in TBS-T containing antibodies for SelR (Abcam, Cambridge, UK), GAPDH (Beyotime Inst. Biotech, Haimen, China), GRP78 (Beyotime Inst. Biotech). After four washes with TBS-T, the blot was incubated for 90 min at room temperature with secondary antibodies diluted at 1:3000 in 5% non-fat milk in TBS-T. Protein bands were identified using an enhanced chemiluminescence (ECL) system (Millipore, Boston, MA, USA). Quantification of protein band in X-ray film was scanned and analyzed with Bio-Rad Quantity One software (Hercules, CA, USA). Protein levels for SelR and GRP78 were normalized to GAPDH.

### 4.7. Measurement of GPx Activity

GPx activity was measured using GPx kit (Jiancheng, Nanjing, China) according to the manufacturer’s protocol.

### 4.8. Measurement of ROS, MDA, PC and TSH

ROS level was assayed using a fluorescence microscope with the fluorescent probe 2′,7′-dichlorodihydro-fluorescein diacetate (DCFH-DA; Beyotime, Haimen, China). For the quantitative analysis of intracellular ROS, cells were collected by trypsinization as described above, incubated with DCFH-DA, and washed three times, then resuspended in serum-free DMEM and DCF fluorescence (excitation, 485 nm and emission, 525 nm) was measured by flow cytometry (FC500, BeckMan Coulter, Brea, CA, USA). The data are presented as the mean fluorescence intensity, and are normalized to the normal control value.

MDA and TSH were detected using measurement kits (Jiancheng, Nanjing, China). The absorbance was measured at 532 nm for MDA and 412 nm for TSH according to the manufacturer’s protocol, respectively.

The protein carbonyl (PC) content was determined by a DNPH assay [53].

### 4.9. Detection of Apoptosis

Cell apoptosis was quantified by flow cytometry (FC500, Beckman Coulter) using an Annexin-V-FITC apoptosis detection kit (Beyotime Inst. Biotech) according to the manufacturer’s protocol [11]. Percentage of early apoptotic and late apoptotic cells was shown in C2 and C4 areas, respectively.

Then nuclear morphology was observed using a fluorescence microscope (IX71, Olympus; Tokyo, Japan). The hLE cells plated in 6-well plates were harvested and washed with PBS, then fixed with methanol and acetic acid (3:1, *v*/*v*) for 20 min at room temperature. After washing, cells were stained with Hoechst 33258 (Sigma) for 30 min at room temperature.

### 4.10. Assay of Cell Caspase-3 Activity

The activity of caspase-3 was assayed using the caspase-3 activity assay kit (Beyotime Inst. Biotech) according to the manufacturer’s protocol. The samples were measured with a microplate reader (Molecular Devices Spectramax 384, San Francisco, CA, USA) at 405 nm and the data were normalized to the control value [54,55].

### 4.11. Detection of Mitochondrial Membrane Potential (Δψm)

The integrity of the mitochondrial membrane potential (Δψm) was evaluated using a cationic dye, JC-1 (5,5′,6,6′-tetrachloro-1,1′,3,3′-tetrabenzimidazole-carbocyanine iodide) (Beyotime Inst. Biotech). Briefly, cells were collected by trypsinization as described above. Then cells were incubated in JC-1 (5 μg/mL, dissolved in serum-free medium) for 30 min at 37 °C. After washing with PBS, the mean green fluorescence (FL-1 channel) and mean orange-red fluorescence (FL-2 channel) were quantified using flow cytometry (BD™ LSR II, San Jose, CA, USA). Δψm was expressed as the ratio of mean red to mean green fluorescence and [56] normalized using normal control value.

### 4.12. Release of Cytochrome c from Mitochondria

The cytochrome c release from the mitochondria was detected uing a human cytochrome c ELISA Kit (R & D Systems, Inc., Minneapolis, MN, USA). The mitochondrial and cytosolic fractions of hLE cells (~1 × 10^7^ cells per sample) were separated using a mitochondria/cytosol fractionation kit (Beyotime Inst. Biotech). The purity of mitochondrial and cytosolic fractions was detected by measuring coxiv and actin protein expressions using Western blot [57,58]. The concentration of protein was determined using the Bradford assay. The cytochrome c concentration (ng/mg of total protein) was determined using the ELISA kit. The data were expressed as the ratio of cytosolic-to-mitochondrial cytochrome c concentrations [59,60].

### 4.13. Statistical Analysis

Representative results from at least three independent experiments are shown as the mean ± the standard deviation. Data analysis was performed by one-way analysis of variance. For comparison of two groups, Student’s *t* test was used. *p* < 0.05 was considered statistically significant.

## 5. Conclusions

In summary, both d-galactose and *SelR* gene knockdown by siRNA independently aggravate the oxidative damage in hLE cells; furthermore, the *SelR* gene knockdown followed by d-galactose treatment could significantly result in ER stress and increase apoptosis accompanying by a decrease of Δψm and a release of mitochondrial cytochrome c, indicating that mitochondrial dysfunction occurred in hLE cells. The results suggest that SelR may play important roles in protecting hLE cell mitochondria against oxidative damage and inhibiting the d-galactose-induced oxidative stress-induced apoptosis in hLE cells by scavenging ROS. However, the role of selenium protecting hLE cells against apoptosis by regulating redox balance is not only via SelR. So it is necessary to do more studies on the nutritional biochemistry of selenium in lens.

## Supplementary Materials

Supplementary materials can be found at http://www.mdpi.com/1422-0067/17/2/231/s1.

## Abbreviations

cDNA	Complementary DNA
DCFH-DA	2′,7′-Dichlorodihydro-fluorescein diacetate
DMEM	Dulbecco’s modified Eagle’s medium
ECL	Enhanced chemiluminescence
ER	Endoplasmic reticulum
FITC	Fluorescein isothiocyanate
GPx	Glutathione peroxidase
GRP78	Glucose-regulated protein
hLE cells	Human lens epithelial cells
JC-1	5,5′,6,6′-Tetrachloro-1,1′,3,3′-tetraethyl-imidacarbocyanine iodide
MDA	Malondialdehyde
MetO	Methionine sulfoxide
Msr	Methionine sulfoxide reductase
MTT	3-(4,5-Dimethylthiazol-2-yl)-2,5-diphenylte-trazolium bromide
NCS	Newborn calf serum
PBS	Phosphate-buffered saline
PC	Protein carbonyl
PI	Propidium iodide
ROS	Reactive oxygen species
SelR	Selenoprotein R
siRNA	Short interfering RNA
TBS-T	Tris-buffered saline with Tween
TSH	Total mercapto group
Δψm	Mitochondrial membrane potential
