# Supplementary material for: Selenoprotein R Protects Human Lens Epithelial Cells against d-Galactose-Induced Apoptosis by Regulating Oxidative Stress and Endoplasmic Reticulum Stress
Source: Int J Mol Sci. 2016 Feb 10;17(2):231. doi: 10.3390/ijms17020231 (PMC4783963; doi:10.3390/ijms17020231)
Supplement: Supplementary file 1 [file ijms-17-00231-s001.pdf]

Supplementary Materials: Selenoprotein R Protects Human Lens Epithelial Cells against D-Galactose-Induced Apoptosis by Regulating Oxidative Stress and Endoplasmic Reticulum Stress

Jie Dai, Hongmei Liu, Jun Zhou and Kaixun Huang *

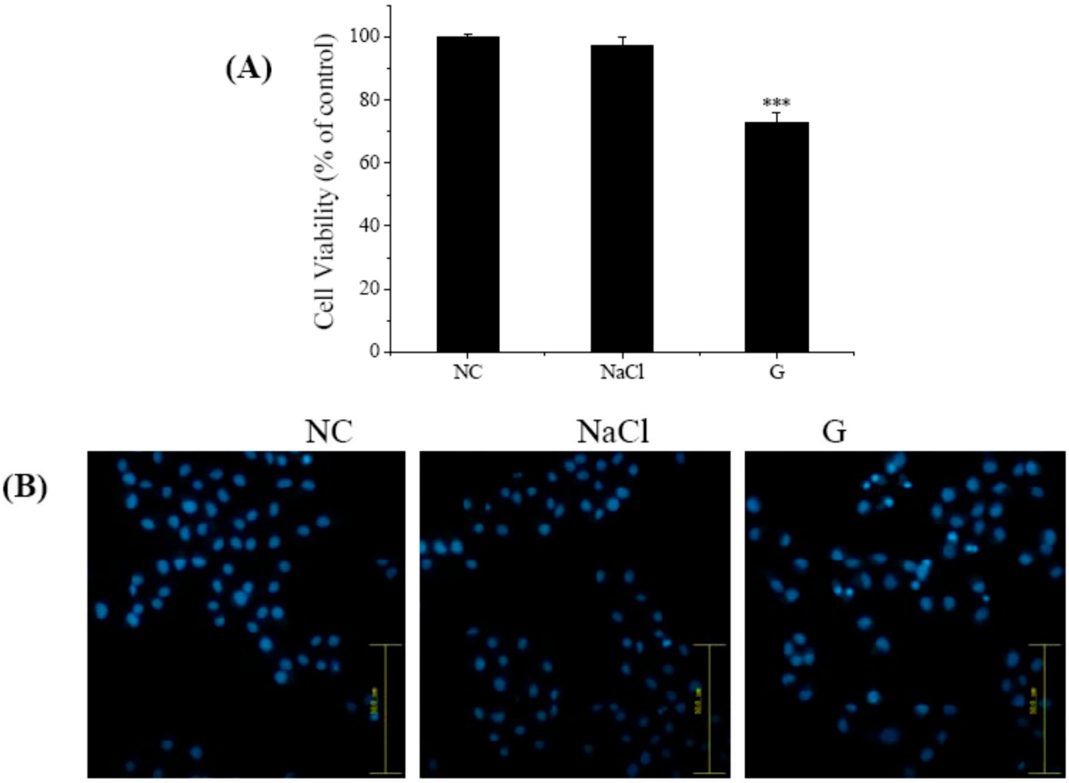

Figure S1. Cell viability investigated using the MTT assay and cell apoptosis observed from cell morphological alterations under the fluorescence microscopy after staining with Hoechst 33258. Data are the mean ± SD of at least three independent experiments. *** $p < 0.001$, compared to the normal control group. NC: normal control cells; NaCl: cells exposed to 75 mM NaCl for 36 h; G: cells exposed to D-galactose (150 mM) for 36 h.
